# Reproductive cycle and gonadal output of the Lessepsian jellyfish *Cassiopea andromeda* in NW Sicily (Central Mediterranean Sea)

**DOI:** 10.1371/journal.pone.0281787

**Published:** 2023-02-14

**Authors:** Marta Mammone, Mar Bosch-Belmar, Giacomo Milisenda, Luca Castriota, Mauro Sinopoli, Alessandro Allegra, Manuela Falautano, Teresa Maggio, Sergio Rossi, Stefano Piraino

**Affiliations:** 1 Dipartimento di Scienze e Tecnologie Biologiche ed Ambientali, DiSTeBA, University of Salento, Lecce, Italy; 2 Laboratory of Ecology, Department of Earth and Marine Science (DiSTeM), University of Palermo, Palermo, Italy; 3 Department of Integrative Marine Ecology (EMI), Stazione Zoologica Anton Dohrn, Palermo, Italy; 4 Department for the Monitoring and Protection of the Environment and for the Conservation of Biodiversity, Italian Institute for Environmental Protection and Research, Palermo, Italy; 5 GRAM Gruppo di Ricerca Applicata al Mare Soc. Coop., Palermo, Italy; 6 CoNISMa, Consorzio Nazionale Interuniversitario per le Scienze del Mare, Rome, Italy; 7 National Biodiversity Future Center (NBFC), Palermo, Italy; University of California Irvine, UNITED STATES

## Abstract

Knowledge of the reproductive strategy is a key prerequisite to predict population dynamics and potential invasiveness of both native and non-indigenous outbreak-forming species. In 2014 the Lessepsian upside-down jellyfish *Cassiopea andromeda* reached the harbor of Palermo (NW Sicily, Thyrrenian Sea), to date its established westernmost outpost in the Mediterranean Sea. To predict *C*. *andromeda* reproductive success in its novel habitat, gonad histology was carried out to record the number and size of mature and immature oocytes. Both male and female simultaneously presented gametes at all stages of development suggesting an asynchronous, yet apparently continuous, reproduction strategy. Indeed, oogenesis was observed throughout the year from pre-vitellogenic, vitellogenetic, and late-vitellogenetic to mature oocytes suggesting multiple reproductive events, as known in other Mediterranean Rhizostomeae. Oocytes were found from May to December, with two seasonal peaks of abundance (late spring = 392 and autumn = 272), suggesting imminent spawning events. Further, jellyfish size varied significantly throughout the year, with maximum diameter (up to 24 cm) in summer, and minimum diameter (6 cm) in winter. Small-sized jellyfish in winter belong to the new cohort, most probably arising from intense summer strobilation of polyps. Late spring fertilization, planula development, and metamorphosis, followed by polyp strobilation in the summer months, may explain the late appearance of a new jellyfish cohort, likely coincident with that recorded throughout winter.

## Introduction

Alien species are considered nowadays one of the major threats for biodiversity and ecosystem functioning [1]. The Mediterranean Sea is considered a hotspot of marine bioinvasions [2, 3] where physical and environmental conditions vary greatly at geographical scale, making possible the entrance of non-indigenous species (NIS) from the Red Sea and the Atlantic Ocean following anthropogenic and natural dispersion patterns [4, 5]. Introduction of marine NIS in the Mediterranean Sea has been facilitated by ships’ fouling and ballast water, aquaculture activities, aquarium trade, and particularly by the progressive enlargement of the Suez Canal [6, 7]. Global warming and species translocation are driving the so called tropicalization of the Mediterranean Sea, with the increasing occurrence of warm-water biota in the basin [8]. On March 2022, 751 validated NIS were considered established and spreading westward, especially from the Levant Sea [9, 10].

Generally, these non-indigenous species become invasive, severely impacting local biodiversity by displacing native species and altering community and ecosystem structures and functioning. As a consequence, such perturbations may transform the ecosystem services and human well-being [11–15]. NIS may have also positive effects on ecosystem functioning through the creation of novel habitats (acting as ecosystem engineers), providing food and shelter to other species or helping in the regulation of the ecosystem functioning in stressed or degraded habitats [14, 16]. The balance between positive and negative effects is sometimes complicated to calibrate, being some features such as the biological cycle, key questions that may help to elucidate such problems.

Cnidarians include a non-neglectable group of new colonizers of the Mediterranean basin [17], whose impact depend both on the species traits and the ecological features of the new habitat [18]. Changes in the relative abundance of jellyfish have socio-economic consequences on coastal human activities and on ecosystems functioning [19–23] by exerting top-down and bottom-up controls on the marine food web, through competition and predation relationships with coexisting organisms [24–27]. *Cassiopea andromeda* (Rhizostomeae, Scyphozoa) is a photosymbiotic tropical jellyfish, originally described from the Red Sea (Forskål 1775) that has invaded the Mediterranean basin. Outside the Mediterranean, it has been recorded across the Indo-Pacific and Atlantic waters but considered as NIS in Hawaii [28] and Brazil [29]. It has an epibenthic (so called upside-down) lifestyle, and it is commonly found lying on seagrass beds, mangrove habitats and lagoons [30, 31]. First discovered in Cyprus in 1903 [32], *C*. *andromeda* colonized the entire Levant Sea [33–35], and spread over the Central Mediterranean in 2009, approaching the Maltese islands [36] and, in 2014, it was found for the first time in Italian waters, specifically in the Palermo “Cala” Harbour, in Sicily [37, 38]. Temperature and several environmental parameters in the Mediterranean Sea are markedly different from tropical seas; nonetheless, *C*. *andromeda* seems to be rapidly spreading thanks to its high asexual proliferative potential through planuloid bud production [39], wide thermal tolerance (18–29°C), and physiological adaptive plasticity to changes of nutrient and light conditions [29, 40–42]. *Cassiopea* spp. in their type localities are mostly associated with mangrove-dominated habitats [28]. Shallow-water anthropogenic areas, such as small bays and harbors (characterized by high nutrient concentrations and high temperature) might facilitate their spread, size and recurrent outbreaks [41, 43–46], supported by plastic symbionts that maintain a high photosynthetic efficiency even when facing a rapid change in light exposure [42]. Available evidence shows that outbreaks (>10 jellyfish/m^2^) of *Cassiopea* can lead to variations in the community structure, e.g., reducing the seagrass abundance and the meadows faunal densities [47]. In addition, *Cassiopea* can locally modify nutrient cycling through ammonium absorption and phosphate uptake [48, 49]; thus, investigating jellyfish biology and ecology may become an essential element for a sound ecosystem management.

One of the main features defining a successful biological invader is represented by its reproductive strategy [50]. A combination of traits, like high fecundity, rapid sexual maturation, high reproductive output, and asexual reproduction and hermaphroditism can sustain such successful invasion [50]. Jellyfish can use different strategies to cope with environmental variability, reduce the risks of local extinction, and opportunistically capitalize into population outbreaks [51] such as: a) species with a complex (planula-polyp-medusa) life cycle, employ a combination of a single, seasonal sexual reproduction event and of multiple, alternative modes of asexual reproduction and overwintering stages [39, 52–54]; b) species with holoplanktonic life cycle with continuous reproduction [55, 56]. Global warming and the rise of seawater temperature may boost jellyfish reproduction, through increased strobilation or budding formation [20] or extend the timing of gonad maturation [56]. This fact, combined especially with overfishing, boosted the medusae proliferation in many areas of the world, including the Mediterranean Sea [20].

In spite of its distinctive symbiotic association and prevalently epibenthic lifestyle, *C*. *andromeda* shares with most Scyphomedusae a typical three-stage life cycle (planula larva, polyp, medusa) and gonochorism [57–59]. Males of *C*. *andromeda* freely release sperms in the water and fertilization take place in the gastrovascular cavity of females, which will store embryos in special brooding vesicles in the center of the oral disc [59, 60]. Nearly 3–4 days post fertilization, competent ciliated planula larvae are released, actively searching for suitable settlement sites, where metamorphosis into the benthic polyp stage eventually occurs [60–62]. Budding of free-swimming planuloids or lateral polyps, and strobilation, are asexual processes regulated by external factors as temperature, salinity, and symbiont presence [62–64]. Whereas bud morphogenesis, settlement, and metamorphosis of *C*. *andromeda* have been widely assessed in scientific literature [65, 66], the detailed characterization of the temporal pattern of sexual reproduction is still missing. In this framework, classical morphometric and histological methods were used here to investigate gametogenesis of *C*. *andromeda* over an annual cycle, by recording the number and size of differentiating oocytes in female gonads. This study was carried out in NW Sicily, i.e., so far, the westernmost location of an established *C*. *andromeda* population in the Mediterranean Sea. Filling this information gap might be important to forecasting further spread of the upside-down jellyfish, especially in recently invaded habitats, and for a better understanding of the mechanisms of outbreaks formation.

## Materials and methods

### Sample collection and processing

Specimens of *Cassiopea andromeda* were sampled by hand net in the Cala harbor of Palermo (NW Sicily; 38° 07.22′ N, 13° 22.09′ E) [46], from 15^th^ of May 2017 to the 20^th^ of April 2018, for a total of 16 collecting dates. Measurements of surface water temperature and salinity were taken at every sampling date with a multiparametric probe (Hanna, HI98194) (S1 Table). Jellyfish were transported to the laboratory and morphological analyses were immediately performed. Diameter of each organism was recorded, and the jellyfish bell was cut off and fixed in 4% formaldehyde solution in seawater. One week before the histological analyses, samples were washed (5 times with non-filtered seawater and once/twice with fresh water) and stored in ethanol 70%.

No permits were required because the sampling site is part of the public maritime domain. Moreover, samplings were carried out by boat and members are either members of the national environmental authority (ISPRA) belonging to the Italian Ministry of Environment, or to bodies of the Ministry of Research and Education, such as the Stazione Zoologica and the universities of Palermo and Lecce.

### Histological analysis

Specimens (n = 64: 16 males and 48 females) of *Cassiopea andromeda* were dissected under a stereomicroscope (Leica MZ6). Two pieces of gonads per organism (total 128 gonads pieces) were cut away and dehydrated using a series of ethanol with increasing concentrations (80% to 100%), cleared in Xylene (histological grade) and impregnated in BioPlast paraffin (Bio-Optica, melting point 56–58°C). The tissue was then embedded in paraffin, sectioned at 7 μm with a Leica microtome (RM 2155), and stained with hematoxylin and eosin. Histological sections were examined under Zeiss Primo Star optic microscope equipped with ZEN software.

### Oocytes count and measurement

Five sections per piece of female gonad was used for oocytes count, 10 sub-replicates per each specimen (n = 48), with a total of 470 examined sections. For female specimens, all visible oocytes were counted with a manual counter. Each count was made in ⁓15 mm^2^ of the ovary. In each section, 10 oocytes were measured along a linear transect (100 oocytes per specimen), choosing a random starting point in the field of view of the microscope. The mean diameter (d) was calculated according to Szafranski et al. [67] by using the following formula:

$$d=\sqrt{l∙w}$$

where “l” is the length of oocytes (i.e., the major diameter) measured in (μm), and “w” is the width of oocytes (i.e., minor diameter) measured in μm.

In the case of male specimens (n = 16), spermatic sacs were counted in 10 sections (five per piece of gonad) per specimen and the diameter of the sacs was also measured.

### Statistical analysis

Temporal pattern and autocorrelation of jellyfish diameter were tested using a generalized additive mixed model (GAMM) with an autoregressive moving average (ARMA) structure to characterize the trend and autocorrelations within the time series. Moreover, as different portions of the gonad were analyzed for each individual, a random effect structure on jellyfish specimens’ ID was introduced to avoid individual pseudo replication problem [68]. The same statistical structure was used to analyzed variation in oocytes number and diameter with jellyfish bell diameter. The validation of the used models was based on the analysis of the normality of the residuals, on the absence of trends in the residuals, and on the inspection of the correlograms, through the ACF function. All analyses were undertaken in R statistical software (R Core Team 2020) using the car [69], mgcv [70], and forecast [71] packages.

## Results

### Oogenesis and spermatogenesis

**Fig 1:**
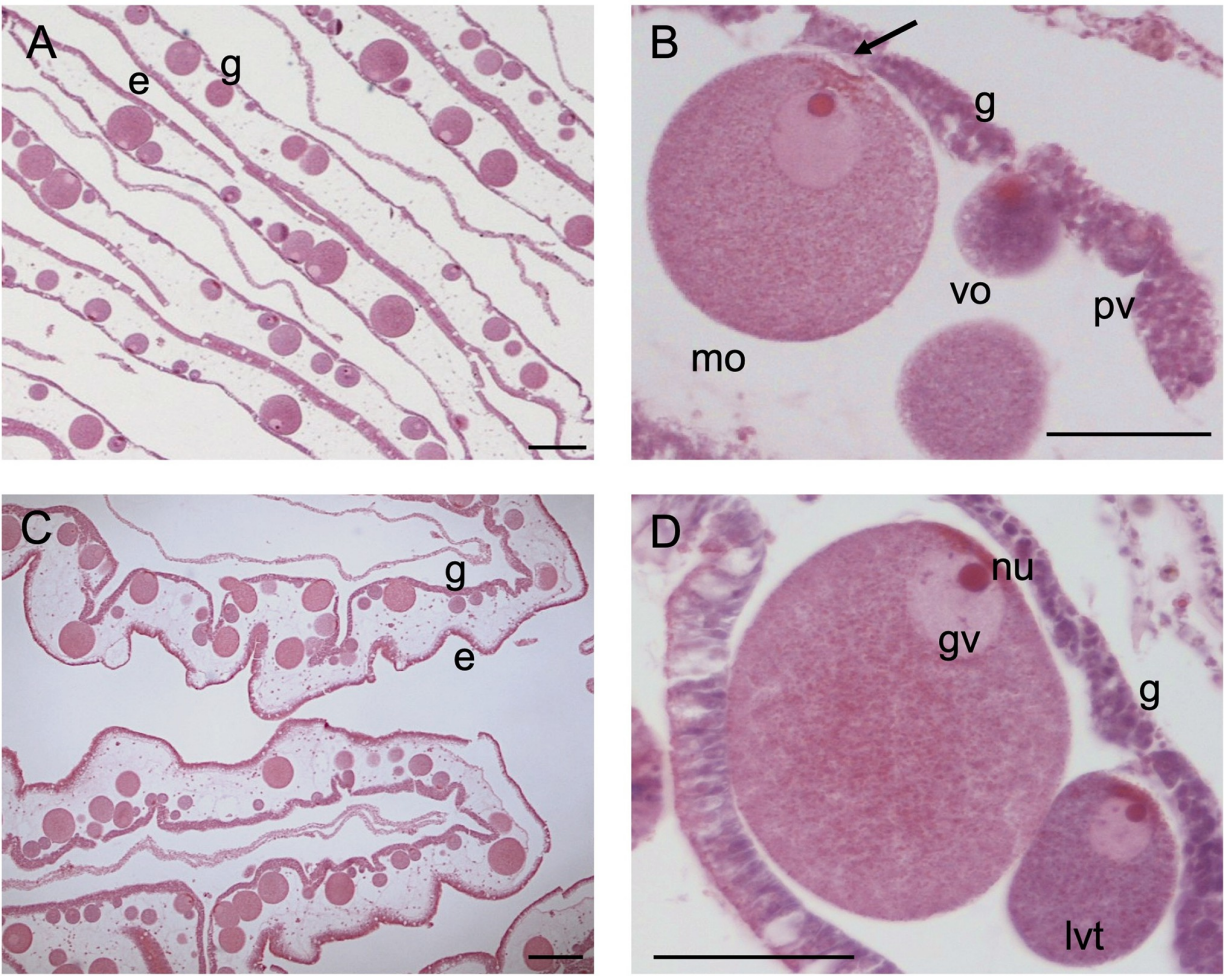
Histological section of a female gonad. (A, C) Ovary (B, D) Development of oocytes. **g** gastrodermis, **e** endodermis, **pv** pre-vitellogenic oocyte embedded within gastrodermis, **vo** vitellogenic oocyte, **lvt** late-vitellogenic oocyte **mo** mature oocyte, **arrow** residual linkage with trophocytes (paraovular body), **nu** nucleolus, **gv** germinal vesicle. Scale bars: A, C = 100 μm; B, D = 50 μm.

Female germ cells (oogonia) rise from the gastrodermis (Fig 1A). Oogenesis starts with the mitotic division of oogonia that will then develop in pre-vitellogenic oocytes (pv) (Fig 1B). Pre-vitellogenic oocytes are still embedded in the gastrodermis and are characterized by a round shape with a darker ooplasm in histological sections, due to the presence of free ribosomes causing basophilia. During development, oocytes increase their size by accumulating yolk in the ooplasm and are progressively displaced into the mesoglea (Fig 1A and 1C). Vitellogenic oocytes are characterized by a round shape, intermediate level of basophilia, some yolk granules and a conspicuous germinal vesicle (that face the gastrodermis) containing a nucleolus. When oocytes have reached ⁓60 μm they are almost entirely embedded in the mesoglea, but always maintaining direct contact with the gastrodermis. Late-vitellogenic oocytes are characterized by an increased yolk granules content that makes them lighter in histological sections and a large germinal vesicle (Fig 1D). Oocytes at the final stage of maturation are squeezed out in the genital sinus through a pit where they were attached through specialized gastrodermal cells termed trophocytes [72] (Fig 1B).

Based on diameters, oocytes were grouped in 4 size classes: <20 μm (oocytes in pre-vitellogenesis), 20–60 μm (oocytes in vitellogenesis), 60–100 μm (late-vitellogenic oocytes), >100 μm (mature oocytes)

**Fig 2:**
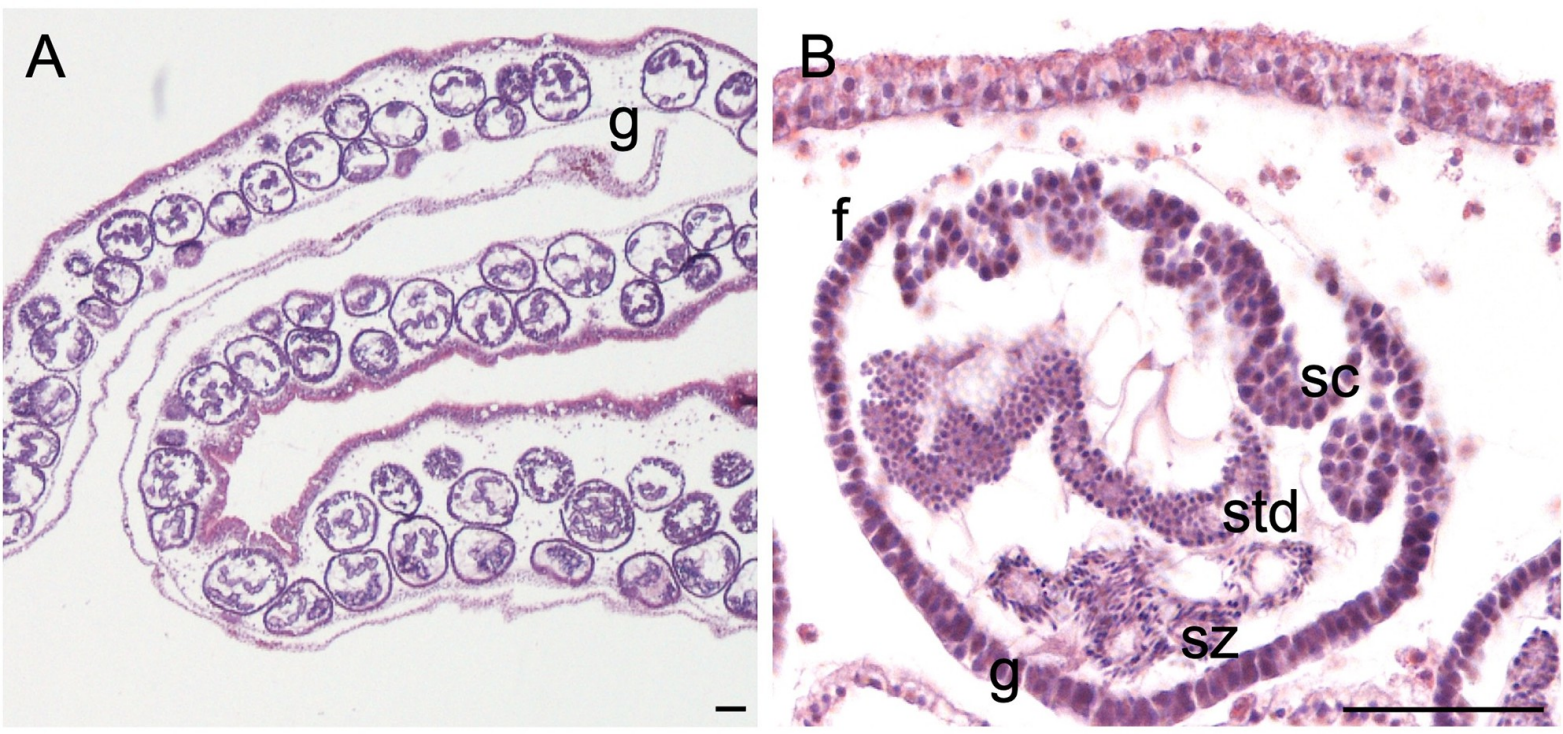
Histological section of a male gonad. (A) Structure of testis (B) Sperm follicle. **f** follicle, **sc** spermatocytes, **std** spermatids, **sz** spermatozoa, **g** gastrodermis. Sperm follicles ranged 93–115 μm during the sampling period. Scale bar = 50 μm.

Spermatogenesis occurs inside follicles, which are distributed evenly in the male gonads (Fig 2A). The process starts from male germ cells (spermatogonia) that going through multiple mitosis give rise to the sperm follicles that have mostly a round/ovoidal shape. Spermatogonia will then develop into primary spermatocytes which will move inward in the follicle. Through meiosis, secondary spermatocytes will be formed, characterized by a high chromatin quantity. With the second meiosis, secondary spermatocytes will form spermatids that will then develop a flagellum and become spermatozoans (Fig 2B). At the final stage of spermatogenesis, the aboral part of the follicle (facing gastrodermis) will be filled by mature spermatozoan with a visible flagellum, packed in sperm bundles called spermatozeugmata [73] (Fig 2B). At this point, they will be released through a pore in the genital sinus and then in the water column. Once male has spawned, follicles are characterized by free space in the lumen and the beginning of a new spermatogenic cycle underline by the presence of new cellular divisions in spermatogonia (Fig 2).

### Temporal pattern of reproduction

**Fig 3:**
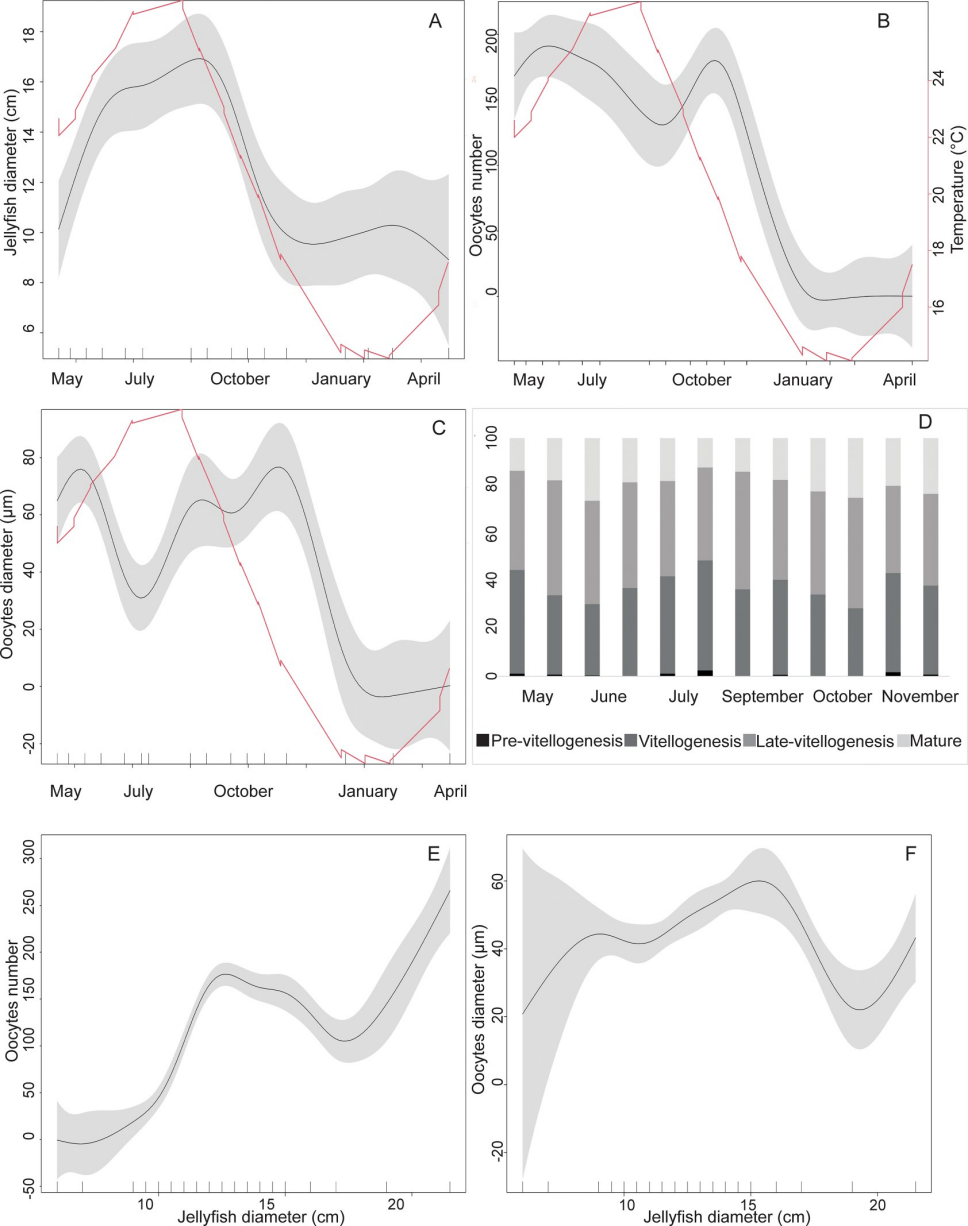
Representation of the temporal pattern of reproduction. (A) Jellyfish diameter (in cm) during the sampling period from May to April (B) Oocytes number per section area (mm^2^) during the sampling period showing two seasonal peaks (late spring and autumn) and seasonal temperatures (C) Oocytes diameter during the sampling period and seasonal temperatures (D) Percentage of each oocyte maturation stage during the whole sampling period. There are two columns for each sampling date (E) Oocytes number in relation to the jellyfish diameter (F) Oocytes diameter in relation to jellyfish diameter. Graphs A, B, C, E, F: The black line represents the mean value while grey areas represent the confidence interval. Temperature is plotted in red.

Sampled jellyfish ranged 6–21.5 cm in bell diameter, changing significantly (Table 1). The largest jellyfish were observed in summer months (> 16 cm diameter) while from December to April, the jellyfish diameter was consistently below 10 cm (Fig 3A).

**Table 1:** Statistical result table. To the left of the tilde (*~*) symbol the response variable, and to the right the independent variable. "s" = natural cubic spline smoother; p = autocorrelation term (AR); q = moving average term (MA); med (medusa ID) and gon (gonad ID nested in med) used as random intercept. Jellyfish_D = jellyfish diameter.

Formula	p	q	random part	Fvalue	pvalue
Oocyte Diameter ~ s(time)	1	2	med = ~1; gon = ~1	27.43	<0.001
Oocytes number ~ s(time)	1	1	med = ~1; gon = ~1	31.38	<0.001
Oocyte Diameter ~ s(Jellyfish_D)	1	2	med = ~1; gon = ~1	5.157	<0.001
Oocytes number ~ s(Jellyfish_D)	1	1	med = ~1; gon = ~1	21.35	<0.001
Jellyfish Diameter ~ s(time)	1	1		10.52	<0.001

Female gonads contained oocytes from May to December, in all stage of development. Two oocytes abundance peaks can be observed over the year, the first in spring-summer (June) and the second one in autumn (October), with a maximal number of oocytes of 392 and 272 (per 15 mm^2^) respectively, while from January to April gonads were empty (Fig 3B). Oocytes ranged from pre-vitellogenic (<20 μm) to mature (>100 μm). Gonads were prevalently filled with pre-vitellogenic and vitellogenic oocytes in July and November (Fig 3C and 3D), and with mature oocytes in June and at the end of October (Fig 3D).

Smaller jellyfish (<10 cm) were found with empty gonads (Fig 3E, S1 Fig); when the bell diameter ranged between 10 and 15 cm the number of oocytes increased and subsequent stabilized, while larger specimens (>17 cm) showed the highest number of oocytes (>100/15mm^2^). The mean oocyte diameter increased when jellyfish bell diameter ranged between 10 and 15 cm, while when jellyfish diameter was between 15 and 20 cm, oocytes diameter dropped (down to 14 μm) (Fig 3F).

### Changes in water temperature and salinity

During late spring (in May) sea water temperature was 22.3°C and increased to 26.35°C at the end of July and up to 26.65 early September. Autumn and winter were characterized by cooler temperatures: with 17.7°C at the end of November and 14.45° C in January. Temperature raised back to 16.25°C in spring months (April) of the following year (Fig 3B). Salinity ranged from 33.95 to 35.7 throughout the whole sampling period.

## Discussion

In the present study, sexual reproduction and gonadal output of the Red Sea alien jellyfish *Cassiopea andromeda* collected in Sicily (Central Mediterranean Sea) were investigated through histological analysis. Specimens sampled in the Palermo harbor showed to be strictly gonochoric over the year, as previously reported from the Red Sea [59]. In contrast, *C*. *andromeda* medusae showed the potential for a transient simultaneous hermaphroditism in an artificial coastal lagoon in the Hawaii islands [73]. The triggering factor for a bisexual gonad maturation in the Hawaii medusae still remains to be clarified. However, given the limited success of sexual reproduction from hermaphroditic medusae, the gonochoric condition seems the most effective reproductive strategy for *C*. *andromeda*, as for most scyphozoans [57].

Both oocytes and sperm follicles were clearly detectable in the gonads of *C*. *andromeda* in mature specimens (size > 10 cm in this study). As in other rhizostome species (*Lychnorhiza lucerna* [74], *Cotylorhiza tuberculata* [75]), oogenesis starts in the gastrodermis, with developing oocytes nourished by gastrodermal trophocytes and, as they increase in size, progressively bulging into the mesoglea. This process is typical for rhizostomes, while absent in coronates except for the deep-sea species *Peryphilla peryphilla* [76, 77]. Fertilization in *C*. *andromeda* is thought to occur within the female gastrovascular cavity that will act as brooding chamber for embryos [59]. As in other rhizostomes, it is hypothesized that oocytes are released outside the ovarian epithelium passing through pits formed in the gastrodermis, possibly where they are in contact with trophocytes [74].

Spermatogenesis occurs inside sperm follicles, as reported for other jellyfish species [57]. No clear timeline of spermatogenesis has been reported for *Cassiopea* but in other rhizostome medusae, as *Nemopilema nomurai*, the entire maturation process (from spermatogonia to spermatozoans) seems to take place–at least—in a couple of days, when jellyfish are kept in a net (at 1m depth) [78]. Rhizostome jellyfish belonging to Kolpophorae (including Mastigiidae, Cepheidae and Cassiopeidae families) have been reported to differentiate sperms clustered by a secretion of lipid nature (spermatozeugmata) [74, 79]. Histological sections of *C*. *andromeda* from Palermo confirm the observation of mature spermatozoans within spermatozeugmata packages (Fig 2).

To analyze sexual differentiation in scyphomedusae, different approaches can be used [56, 80, 81]. Female specimens can be classified as mature or immature based on the presence of post-fertilization sexual products, i.e., as embryos or larvae; differently, the progress of gametogenesis can be investigated morphologically and biochemically, assessing the state of gamete differentiation [52]. In the second case, oocytes are classified by yolk density and diameter (oocytes size distribution) whereas the maturity of male sperm follicles can be assessed by the relative abundance of spermatogonia, spermatocytes, spermatids, and of functional sperms [56, 81]. In the present work, using histological analyses, the morphological approach was applied, with the definition of new size classes (pre-vitellogenic, early vitellogenic, late vitellogenic, and mature oocytes) for oocyte differentiation, specifically applicable to *C*. *andromeda*. Finally, the occurrence of fully developed gametes is regarded as a sexual maturity proxy, related to potentially imminent spawning events [74].

Both male and female specimens collected in Palermo harbor simultaneously possess gametes at different level of differentiation, from early vitellogenic to mature oocytes and from spermatids to mature sperms, suggesting an asynchronous reproduction strategy, as observed in other rhizostomes [53, 82]. From May to December female specimens always had oocytes at different stages of differentiation, including mature oocytes, but these were found with two seasonal peaks of abundance (late spring and autumn), probably linked to the imminence of bimodal spawning events, and suggesting an iteroparous reproduction (as in other outbreak-forming Mediterranean scyphozoans [56, 83]). The same pattern is observed in native Mediterranean jellyfish, such as *Pelagia noctiluca*, characterized by spring and autumn spawning events [56]. Therefore, *C*. *andromeda* can be considered a seasonal species in the Mediterranean, tuning its increasing growth and sexual reproduction with higher water temperature. In fact, larger specimens (diameter > 16 cm) were found in early September, when water reached the highest temperature (26.6°C) and jellyfish showed the highest number of oocytes in the gonads (> 20 oocytes per mm^2^ of gonad tissues), disclosing the achievement of full sexual maturity.

Accordingly, minimum jellyfish bell diameters were found in January. The presence of small jellyfish (< 10cm) from January to April suggests the recruitment of new cohort following a summer strobilation, that can be observed in *Cassiopea* at temperatures > 17–18°C [64] but with optimal condition ≥ 25°C [62]. This is in line with the reproductive strategy of other scyphozoans in the Mediterranean Sea: in late summer, the newly born jellyfish spread into the water column taking advantage of the renewed trophic conditions, after the stratification occurred in the warm period [84].

Oocyte diameter changed over time following the same trend. In particular, the late-spring and autumn peaks were characterized also by a higher proportion of mature oocytes, meaning that by the expected successive fertilization, polyp formation and strobilation a new cohort may arise, likely corresponding to that found during winter months. This is also supported by the observation that, in the winter period, gonads were empty. This observation is also paralleled by a drop in the jellyfish diameter (<10 cm) supporting the hypothesis we deal with new, still immature cohort. The two spring and autumn peaks do not present a difference in term of oocyte diameter, which represent a key factor for animal reproductive strategies [85]. *Cassiopea andromeda* from this study had smaller oocytes than reported by literature (140–170 μm; [59]) but larger oocytes than other rhizostomes as *Rhizostoma pulmo* (personal observations, up to 68 μm for mature oocytes). Oocyte diameter is known to be influenced by both biotic and abiotic factors; for instance, a direct positive relationship exist with temperature [86, 87]. In fact, *C*. *andromeda* oocytes were larger in the warm periods, when the higher temperatures boost oocyte differentiation. Another major driver of gonadal input is the food source availability [84] since sexual reproduction requires a large investment of energy for gonadal development and gamete differentiation [88]. As a mixotrophic species with a flexible nutritional mode, *C*. *andromeda* might be greatly favored when facing changing environmental conditions [42, 45, 50]. In spring, it may take advantage of available seston and organic matter through a classical heterotrophic feeding; in autumn, the jellyfish may use a reservoir of photosynthates produced throughout the high-irradiance summer period, coincident with the reduction of heterotrophic resources. The harbor of Palermo, as other coastal confined habitats in the Mediterranean Sea (like the Salini lagoon in Malta, see [89]), seem to be ideal spots for *C*. *andromeda* establishment and persistence [46]. In fact, these coastal protected environments are characterized by nutrient-enriched, calm waters and a reduced number of predators, compared to the natural, more exposed coastal habitats. This condition may favor the growth and reproduction of *C*. *andromeda*, originally from the protected habitats of mangrove lagoons in the Indo-Pacific area [28]. The high reproductive potential of the upside-down jellyfish, coupled with, and seemingly sustained by, a high nutritional flexibility and photosynthetic plasticity [42], and wide eurythermal tolerance [45], may appoint *C*. *andromeda* as a potential winner in the current warming scenario of the Mediterranean Sea.

## Supporting information

S1 Fig **A** gonads during the winter months **B** close up on gonads containing only germ cells. Scale bar = 50 μm.
(DOCX)

S1 Table Environmental data. Temperature (°C) and salinity data during the sampling period.
(DOCX)
