# Supplementary figures and images for: Reproductive cycle and gonadal output of the Lessepsian jellyfish Cassiopea andromeda in NW Sicily (Central Mediterranean Sea)
Source: PLoS One. 2023 Feb 14;18(2):e0281787. doi: 10.1371/journal.pone.0281787 (PMC9928113; doi:10.1371/journal.pone.0281787)

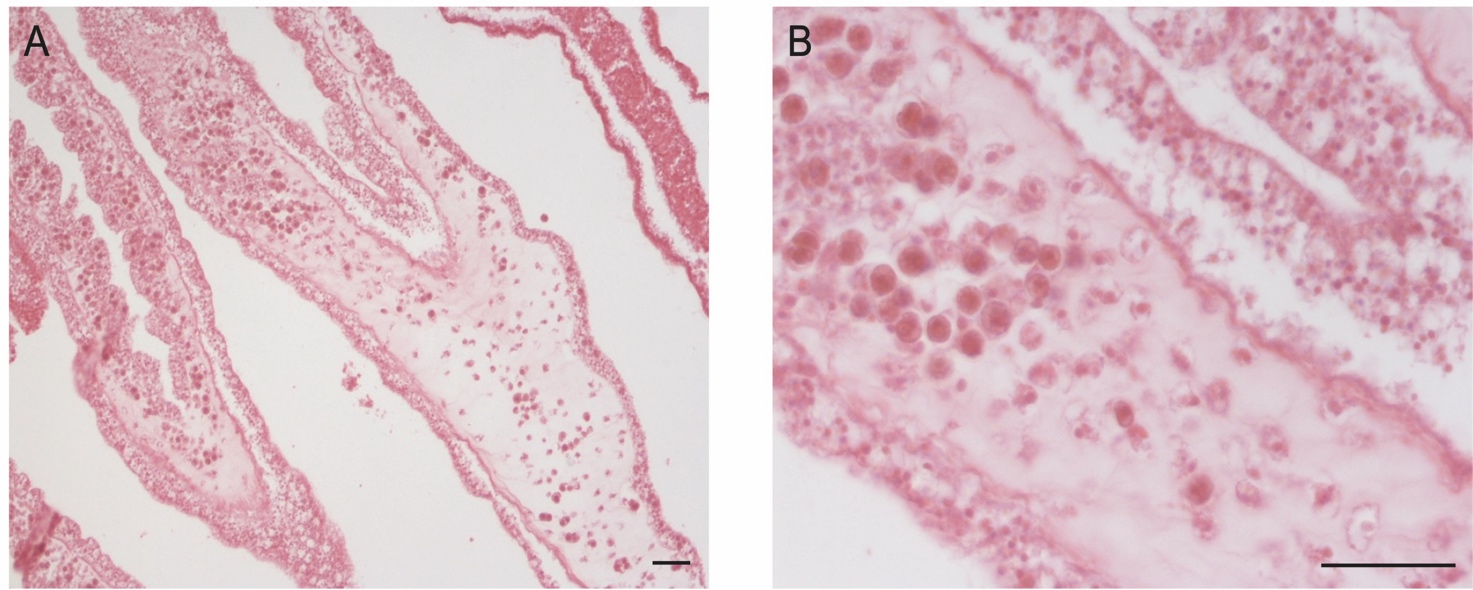

Supplement: S1 Fig — A gonads during the winter months B close up on gonads containing only germ cells. Scale bar = 50 μm. (DOCX) [file pone.0281787.s001.docx]
