# Supplementary material for: Reproductive cycle and gonadal output of the Lessepsian jellyfish Cassiopea andromeda in NW Sicily (Central Mediterranean Sea)
Source: PLoS One. 2023 Feb 14;18(2):e0281787. doi: 10.1371/journal.pone.0281787 (PMC9928113; doi:10.1371/journal.pone.0281787)
Supplement: S1 Table — Temperature (°C) and salinity data during the sampling period. (DOCX) [file pone.0281787.s002.docx]

| Sampling date | Water Temperature (°C) | Salinity |
| --- | --- | --- |
| 05/25/2017 | 22.3 | 33.95 |
| 06/08/2017 | 22.75 | 34.85 |
| 06/22/2017 | 24.05 | 35.1 |
| 07/12/2017 | 25.1 | 35.23 |
| 07/27/2017 | 26.35 | 35.4 |
| 09/07/2017 | 26.65 | 35.45 |
| 09/21/2017 | 25.05 | 35.7 |
| 10/12/2017 | 22.9 | 35.43 |
| 10/26/2017 | 21.25 | 35.4 |
| 11/10/2017 | 19.85 | 35.48 |
| 11/29/2017 | 17.7 | 35.55 |
| 01/19/2018 | 14.45 | 35.35 |
| 02/08/2018 | 14.25 | 35.3 |
| 03/01/2018 | 14.15 | 35.3 |
| 04/20/2018 | 16.25 | 34.6 |
